# TECHNOLOGICAL AND FINANCIAL PREDICTORS OF FEAR OF FINANCIAL ABUSE AMONG OLDER ADULTS

**DOI:** 10.1093/geroni/igz038.1779

**Published:** 2019-11-08

**Authors:** Shaina Alves, Erin Grinshteyn

**Affiliations:** University of San Francisco, San Francisco, California, United States

## Abstract

Much research has focused on elder abuse. Less research focuses on fear of abuse. This analysis examines the associations between feelings of technological competence and variables assessing financial confidence with fear of financial victimization. Data were collected among community dwelling older adults in Nevada (n=467). Questions were asked regarding technological competence, confidence in navigating the financial system, asset protection, trust in financial institutions, and previous financial abuse victimization. The outcome was assessed by asking how afraid the respondent was of becoming a victim of financial abuse. Multivariate logistic regression models were run controlling for confounding. Controlling for all covariates, those who reported feeling unconfident in their technological competence had 2.5 times the odds of being afraid of financial abuse compared with those who felt confident (p<0.02). Those who reported feeling like their assets were at risk had 4.12 times the odds of being afraid of financial abuse (p<0.0001). Older adults who reported feeling vulnerable to financial victimization had 9.4 times the odds of being afraid of financial abuse compared with those who felt invulnerable (p<0.0001). Those who were previously victims of financial abuse had 4.33 times odds of being afraid of financial abuse compared with those who had no history of financial abuse (p<0.0001). Feeling confident in the financial system, asset protection, fear of credit card use, and trust in financial institutions were not associated with fear of financial abuse. These data provide a better understanding of fear of financial abuse, which will allow for better prevention of this issue.

